# Pregnancy and delivery in patients with Charcot-Marie-Tooth disease and related disorders

**DOI:** 10.1177/1753495X221107328

**Published:** 2022-06-16

**Authors:** Mariola Skorupinska, Gita Ramdharry, Bridgette Byrne, Matilde Laurá, Mary M Reilly

**Affiliations:** 1 Centre for Neuromuscular Diseases, 481644 UCL Queen Square Institute of Neurology, University College London, London, UK; 2 8830 Coombe Women and Infants University Hospital, Dublin, Ireland

**Keywords:** Charcot–Marie–Tooth disease, pregnancy, delivery, obstetric complications

## Abstract

**Background:**

Charcot–Marie–Tooth disease is the most common inherited peripheral neuropathy and many patients with Charcot–Marie–Tooth are women of childbearing age. Guidelines for managing pregnancy in Charcot–Marie–Tooth are lacking.

**Aims:**

To assess the impact of pregnancy on Charcot–Marie–Tooth and how Charcot–Marie–Tooth affects pregnancy, delivery and postnatal care.

**Methods:**

A retrospective questionnaire exploring disease course during pregnancy, delivery, pregnancy complications, anaesthetic management and puerperium was administered to 92 patients with Charcot–Marie–Tooth and related disorders.

**Results:**

Worsening of Charcot–Marie–Tooth symptoms were reported in 37% of pregnant patients which resolved after delivery in half of the patients. No significant increase in pregnancy, delivery and anaesthetic complications were observed and the type of delivery did not significantly differ from the normal population.

**Conclusions:**

While these results are reassuring, ideally an international prospective study should be done to confirm these results and to develop practice guidelines on the management of pregnancy in Charcot–Marie–Tooth.

## Introduction

Charcot–Marie–Tooth disease (CMT) and related disorders (hereditary sensory neuropathy (HSN), hereditary motor neuropathy (HMN) and hereditary neuropathy with liability to pressure palsies (HNPP)) are the commonest group of inherited neuromuscular diseases with an estimated prevalence of 1:2500 and 1:10,000.^1,2^

CMT is a clinically heterogeneous disease usually characterised by slowly progressive, length-dependent muscle wasting, weakness, and sensory loss in the upper and lower limbs^3^ leading to balance and gait difficulties.^4^

Disease onset is usually in the first two decades of life and the majority of individuals are diagnosed prior to 30 years of age including many women of childbearing age. In clinical practice, patients frequently ask whether pregnancy will affect their condition, whether CMT will affect their pregnancy, what type of delivery they should have and whether they or their child will have a higher risk of complications during pregnancy and delivery. Current knowledge is based predominantly on case reports and retrospective series^5–8^ and specific guidelines for the management of pregnancy and delivery in CMT are not available.

Conflicting findings have been reported on obstetric complications in CMT patients with higher risk of abnormal presentation, postpartum haemorrhage (PPH) and operative delivery^9^ which were not confirmed by a subsequent international retrospective study.^5^ More recently two large retrospective studies using self-reported questionnaires were performed in Italy^8^ and in Germany.^7^ The Italian study observed a higher rate of placenta praevia, abnormal presentation and preterm deliveries compared to the reference population, whereas the German study did not show any significant differences in terms of obstetric complications compared to their reference population. Moreover, some studies reported worsening of CMT symptoms during pregnancy ranging from 16.3% to 38% of women^5–8^ with a variable rate of persistence of symptoms post-delivery (between 20% and 77%).^5,6,8^

The aim of this study was to retrospectively evaluate the impact of pregnancy on CMT and the risk of complications during pregnancy and delivery in a large cohort of patients in the UK attending a single neurology centre with a particular emphasis on obstetric complications as these differed between the recent Italian and German studies.

## Material and methods

This project was registered as a service evaluation in line with local governance procedures. Participation in this study was voluntary, and all collected data were anonymised and kept confidential.

Retrospective single-site service evaluation was performed using a study survey questionnaire. Patients were invited to participate in the study during their routine clinic visits to the inherited neuropathy clinics in the National Hospital for Neurology and Neurosurgery, London, UK. CMT disease Examination Score version 2 (CMTESv2) was collected as part of their routine visits and the study questionnaire was administered by the research nurse (MS) during the same visit. CMTESv2 is a subscore of the CMT Neuropathy Score (CMTNSv2),^10^ a validated composite scale to assess CMT impairment (0–28 points) which includes patients’ symptoms and signs. A retrospective questionnaire was designed with expert help from an obstetrician (BB) with a special interest in pregnancy in patients with medical conditions, to assess the impact of pregnancy on CMT and to determine how CMT affects pregnancy, the delivery and the postnatal care. The questionnaire was divided into 4 parts (prior, during, after pregnancy and delivery) and included 29 questions on disease impairment, falls, pain, fatigue and respiratory complications before and during pregnancy, type of delivery and possible complications, on anaesthesia and postnatal care. All women attending the inherited neuropathy clinics in the National Hospital for Neurology and Neurosurgery, London, UK between November 2014 and March 2020 with a history of pregnancy were eligible. Data on type of delivery, complications and anaesthesia were compared to the UK population using NHS maternity statistics, England 1980–2019 (https://digital.nhs.uk/data-and-information/publications/statistical/nhs-maternity-statistics/2018-19).

### Statistical analysis

Data collected on type of delivery, anaesthesia or analgesia, pregnancy and delivery complications were compared to national figures (NHS Maternity Statistics, England 1980–2019. All outcomes were binary (yes/no) in nature, and the one sample test of proportions was used for analyses.

Analyses to assess the deterioration of symptoms pre-and during pregnancy as they were in the same group of women on both occasions were paired in nature. All outcomes were binary in nature; the analyses were performed using the paired exact test.

### Results

Between November 2014 and March 2020, 92 women with CMT and related disorders were enrolled in the retrospective study. Two hundred and four pregnancies resulted in 171 births. The majority of women (86/92 (93%)) had a diagnosis of CMT. A very small proportion 2/92 (2%) and 4/92 (4%) had HMN and HNPP, respectively. Of those with CMT 58% (50/86) had CMT1A due to *PMP22* duplication, 15% (13/86) had CMTX due to a *GJB1* mutation, 7% (6/86) had CMT2A due to *MFN2* mutation; the remaining had various subtypes of CMT and related disorders (Table 1). Only two patients did not have a molecular diagnosis (CMT2, CMTi). Impairment, as measured by CMTES v2, was on average 9.77 ± 5.42 (range: 0–28) (Table 1). The average age at the first pregnancy was 28.2 ± 5.4 years (range: 17–41) and average age at completing the pregnancy questionnaire was 40.6 ± 10.4 years (range: 20–70).

**Table 1.** Demographic and clinical data.

Clinical diagnosis	Genetic diagnosis	Number of patients (%)
CMT		86 (93)
CMT1A	Chr 17 dupl	50 (58)
CMT1X	GJB1	13 (15)
CMT2A	MFN2	6 (7)
CMT1B	MPZ	2 (2)
Other CMT		15 (17)
HMN	HSPB1	2 (2)
HNPP	Chr 17 del	4 (4)
		Mean ± SD (range)
CMTESv2^a^		9.77 ± 5.42 (0–28)
Age at first pregnancy		28.2 ± 5.40 (17–41)
Age at completing questionnaire		40.6 ± 10.42 (20–70)

^a^ CMTES was performed at the time of the questionnaire.

CMT, Charcot–Marie–Tooth; Chr 17 dupl, Chromosome 17 duplication, Chr 17 del, Chromosome 17 deletion, CMTESv2, Charcot–Marie–Tooth Examination Score version 2; HMN, Hereditary Motor neuropathy; HNPP, Hereditary Neuropathy with liability to pressure palsies.

## Disease course during pregnancy

Patients reported worsening of CMT symptoms in 36.8% (63/171) of pregnancies which resolved after delivery in 49.2% (31/63) of affected pregnancies (Table 2). Among the symptoms that worsened during pregnancy, walking and balance deterioration were reported in 71.4% (45/63) and 58.7% (37/63) of affected pregnancies, respectively (Table 2). Deterioration was mostly reported in the second and third trimesters of affected pregnancies. Worsening was reported in the first trimester in 16/63 (25%), in the second trimester in 33/63 (52%) and in the third trimester in 36/63 (57%) of affected pregnancies. Some women reported worsening only during one trimester (6/63 (9.5%) in the first, 12/63 (19%) in the second, 14/63 (22%) in the third) and some women during two trimesters (10/63 (16%) first and second, 21/63 (33%) second and third. Deterioration was reported during all trimesters in 10/63 (16%) affected pregnancies.

**Table 2.** Deterioration of symptoms during pregnancy.

	N = 171	%
Symptoms deterioration	63/171	36.8
Walking	45/63	71.4
Balance	37/63	58.7
Symptoms resolution after delivery	31/63	49.2

In addition, patients were specifically asked to compare specific variables pre and during pregnancy (Table 3). The number of falls decreased significantly during the pregnancy from 67.3% (115/171) to 39.8% (68/171) (*p*-value <0.001) and there was no significant increased use of orthosis, walking aids or wheelchair. Pain in the lower limbs (feet, ankles, knees and hips) did not increase significantly.

**Table 3.** Comparison of symptoms and functional impairment before and during pregnancy.

	Pre (N = 171) n (%)	During (N = 171) n (%)	Difference (*) % (95% CI)	*P*-value
Assistance to walk	39 (22.8%)	43 (25.2%)	2.3% (−2.2%, 6.9%)	0.39
Orthoses – Insole/Shoe	19 (11.1%)	18 (10.5%)	−0.6% (−3.7%, 2.6%)	1.00
Orthoses – Ankle/foot	16 (9.4%)	15 (8.8%)	−0.6% (−2.3%, 1.1%)	1.00
Walk. Aid – Unilateral	3 (1.8%)	7 (4.1%)	2.3% (−0.5%, 5.2%)	0.13
Walk. Aid – Bilateral	3 (1.8%)	8 (4.7%)	2.9% (−0.1%, 6.0%)	0.06
Wheelchair – Regular	4 (2.3%)	6 (3.5%)	1.2% (−1.0%, 3.4%)	0.50
Wheelchair – Intermit.	2 (1.2%)	0 (0.0%)	−1.2% (−3.4%, 1.0%)	0.50
Falls	115 (67.3%)	68 (39.8%)	−27.5% (−35.0%, −20.0%)	**<0.001**
Pain	77 (45.0%)	82 (48.0%)	2.9% (−2.9%, 8.7%)	0.38
Fatigue	91 (53.2%)	131 (76.6%)	23.4% (15.3%, 31.5%)	**<0.001**
Respiratory complications	3 (1.8%)	3 (1.8%)		

(*) Differences are reported as outcome during pregnancy minus outcome pre-pregnancy.

Statistically significant values are in bold.

Fatigue was reported in half of the women (53%) before the pregnancy and this significantly worsened during pregnancy (*p*-value <0.001) and was reported in 76.6% (131/171) of pregnancies. Respiratory complications were rarely reported in our cohort of patients and no significant increase in respiratory symptoms was reported during the pregnancy (Table 3).

## Obstetric complications

Miscarriage occurred in 33/204 (16%) of pregnancies, a rate that is in keeping with that reported in the general obstetric population.^11^ All but five were reported before 12 weeks. Five miscarriages were at 12 weeks or later (12–19 weeks).

Complications related to pregnancy were reported in 25% (43/171) of women. Of these, vaginal bleeding before 20 weeks and urinary tract infection were reported in 7% (12/171) and 4%, (7/171) of pregnancies, respectively, which was significantly higher than that of the UK population (Table 4). Other complications such as hypertension or pre-eclampsia, gestational diabetes and pre-term delivery were not different from the background population (Table 4).

**Table 4.** Delivery, anaesthesia and pregnancy-related complications.

	CMT		UK Population	*P*-value
	n	% (95% CI)		
*Type of delivery*	171			
Vaginal delivery	104	61% (53%, 68%)	68.0%	0.05
Assisted delivery	23	13% (8%, 19%)	12.1%	0.73
Caesarean delivery	44	26% (19%, 33%)	20.2%	0.09
*Delivery complications*				
Fetal distress	10	5.8% (2.8%, 10.5%)	26.1%	**<0.001**
Postpartum haemorrhage (PPH)	5	2.9% (1.0%, 6.7%)	20.6%	**<0.001**
Long labour	23	13.5% (8.7%, 19.5%)	19.4%	0.05
*Anaesthesia*	171			
Epidural	49	28.7% (22.0%, 36.1%)	17.7%	**<0.001**
	44	CS delivery		
Epidural	17	38.9% (24.4%, 54.5%)	15.2%	**<0.001**
Spinal	21	47.7% (32.5%, 63.3%)	56.2%	0.29
General anaesthesia	6	13.6% (5.2%, 27.4%)	7.7%	0.15
*Pregnancy-related complications*	171			
Hypertension or pre-eclampsia	9	5.3% (2.4%, 9.8%)	5.3%	0.54
Vaginal bleeding before 20 weeks	12	7.0% (3.7%, 11.9%)	0.02%	**<0.001**
Vaginal bleeding after 20 weeks	4	2.3% (0.6%, 5.9%)	2.1%	0.78
Gestational diabetes	8	4.7% (2.0%, 9.0%)	8.3%	0.05
Urinary tract infection	7	4.1% (1.7%, 8.3%)	0.8%	**0.04**
Preterm delivery	17	9.9% (5.9%, 15.4%)	6.3%	0.10

CS, caesarean section; CMT, Charcot–Marie–Tooth.

Statistically significant values are in bold.

## Delivery and anaesthesia

The average gestation at delivery was 39 weeks (±1.83), range 34–42. The majority of the babies (90% (154/171 pregnancies)) were born at ‘term’ (37 weeks gestation or later), with 10% (17/171 pregnancies) premature (born before 37 weeks) (range: 34–36 gestation weeks). The majority (112/171) of babies (66%) were delivered in the labour unit, 26% (45/171) in theatre, 5% (9/171) at birthing centre/midwife led ward and 3% (5/171) at home.

Slow progress and long labour were reported in 9% (15/171) and 13.5% (23/171) of pregnancies, respectively, which was similar to the UK general population (Table 4). Fetal distress was reported in 5.8% (10/171) and PPH in 2.9% (5/171) which were significantly lower than the UK population (26.1% fetal distress and 20.6% PPH) (*p*-value < 0.001).

The majority of women 61% (104/171) had vaginal births: assisted by forceps or ventouse in 13% (23/171). The remainder had caesarean section (CS) (26%, 44/171). 55% (24/44) were delivered by elective CS. 45% (20/44) had emergency CS during labour due mainly to either slow progress or fetal distress; There were no significant differences in the rate of assisted deliveries or the type of deliveries in comparison with the UK population (Table 4). Vaginal deliveries occurred slightly less in the CMT group, whereas caesarean deliveries were slightly more frequent in the CMT group but the difference from national figures did not reach statistical significance (Table 4).

In terms of anaesthetic management, epidural anaesthesia was performed in 28.7% (49/171) of all deliveries. Of those patients who had CS, 38.9% (17/44 pregnancies) had epidural anaesthesia, 47.7% (21/44 pregnancies) spinal anaesthesia and 13.6% (6/44 pregnancies) general anaesthetic. An epidural provided anaesthesia for 13/23 assisted deliveries (56%) and 19/104 natural deliveries (18%). There was no significant difference in the use of general anaesthesia, spinal and combined spinal and epidural anaesthesia in the CMT cohort in comparison with the UK population; however, the use of epidural anaesthesia in all deliveries was significantly higher in CMT women (Table 4). Five women (six pregnancies) reported some complications related to anaesthesia (four epidural, two spinal) which were described as delayed anaesthetic effect in two patients or prolonged anaesthetic effect in one patient. One patient reported difficulties with epidural administration because of scoliosis and one reported low blood pressure and loss of consciousness for a few seconds during spinal anaesthesia.

## Post-pregnancy period

During the post-partum period, difficulties looking after the newborn baby were reported in 27% (47/171) of patients. Patients mainly described difficulties carrying the baby (60% (28/47)), hand difficulties (43% (20/47)) and walking difficulties (38% (18/47)). The majority of patients (74% (35/47)) needed help from the partner and relatives.

## Discussion

This retrospective study was performed in a single UK genetic neuropathy centre ensuring accurate genotype and phenotype data including the CMTESv2 being done by clinicians experts in CMT. The study questionnaire was administered by a research nurse during the clinic visit to make sure patients understood the questions in order to collect accurate responses. In our cohort, the vast majority of patients (94%) had CMT with more than half having CMT1A which is the most common form of CMT. Only a small proportion of patients had either HMN or HNPP and none had HSN. Our data showed worsening of symptoms in 36.8% of pregnancies, with resolution of symptoms after delivery in 49.2% of affected pregnancies. This is similar to other studies^5,6,9^ where the percentage of worsening varied between 32% and 38% but it differs from a recent large retrospective Italian study where worsening of CMT symptoms was reported only in 16.3% of patients.^8^ A likely explanation for the difference between the Italian and the UK studies is the way the studies were done as the Italian study was an online multicentre self-reported questionnaire, whereas the UK study was a single centre study with the questionnaire being administered in person during a clinic visit.

In our cohort, the symptoms most commonly reported to deteriorate during pregnancy were related to walking and balance. As this worsening was more commonly reported in the last two trimesters, a possible explanation could be due to changes related to pregnancy (e.g. weight gain and change of centre of gravity). However when asked specific questions regarding functional impairment (use of orthosis, walking aids) and symptoms before and during pregnancy, there was no significant increase in the need for walking aids apart from a small increase in the percentage of patients requiring unilateral or bilateral walking assistance which did not reach statistical significance. Interestingly, there was a significant reduction in the number of falls during pregnancy which may be due to patients being more careful when walking. These data are difficult to compare with some of the previous studies as these specific aspects were not included.^5,7^

In our cohort, half of the patients reported resolution of symptoms after delivery, whereas no recovery after delivery in the majority of pregnancies was observed in the Italian registry.^8^ This difference is likely to be related to the difference in both the questions asked and the way the study was done and underlines the need for a future prospective study.

We assessed separately fatigue as a symptom as this is commonly described during the course of pregnancy. In our cohort, more than half patients reported fatigue before the pregnancy and this significantly worsened during gestation similarly to the Italian study.

In our cohort, there was no significant increase in pregnancy, or delivery-related complications in comparison with the control population. The rate of miscarriage, bleeding after 20 weeks, hypertension/pre-eclampsia and gestational diabetes was no different to controls (Table 4). There was a higher rate of vaginal bleeding before 20 weeks and urinary tract infection (7% and 4%, respectively) in the CMT population but these early pregnancy complications may not be captured in the national database and are more prevalent in observational studies. Vaginal bleeding before 20 weeks of gestation and UTI are reported to complicate approximately 20%–25%^12^ and 8%^13^ of normal pregnancies respectively. There was no increased occurrence of malpresentation as reported by Hoff et al.^9^ or placenta praevia as reported by Pisciotta et al.^8^

The mean gestation at delivery was 39 weeks. Preterm delivery (9.9%) was no different from the UK population, but was reported to be increased in the Italian study, at a rate of 17.5% when twins were excluded.^8^ Preterm birth is estimated to occur in 5%–18% of pregnancies worldwide.^14^

The type of delivery in CMT patients did not differ significantly from the normal population. Importantly, we did not find an increase in operative vaginal delivery (OVD) rates reported by Hoff et al.^9^ with a rate of 13% compared to the national data (12%). This finding is supported by a recent large study^8^ where only one woman with CMT had an OVD, but it is difficult to comment on this outcome in this study when the background rate of OVD is low.^15^ The rate of CS, however, does not differ from the control populations in our study and recent studies.^7,8^

Our study showed no clear evidence of complications related to anaesthesia with only five patients reporting either delayed or prolonged anaesthetic effect. One patient reported difficulties with siting of an epidural related to scoliosis, a not uncommon feature of CMT. Regional anaesthesia in patients with neuromuscular diseases has been controversial, in the past, and even suggested as a risk factor for nerve damage.^16^ Currently, there is scarce evidence on the optimal choices of anaesthesia in CMT. No controlled studies have evaluated the risk of general or regional anaesthesia in the condition^17^ but there is no published evidence that epidural or spinal anaesthesia are contraindicated. There has been a previous report from 1987 on slower but uncomplicated recovery in CMT patients having general anaesthesia for caesarean delivery.^18^ General anaesthesia carries greater risk in pregnant women,^19^ especially in an emergency situation, such that regional anaesthesia is preferred for CS in the general population. The lack of consensus and uncertainty about anaesthetic management in CS in CMT is reflected in the reported use of general anaesthesia in recent studies (13% in our study; 19.4% Rudnik; 60% Pisciotta). In our study, we observed a higher use of epidural anaesthesia during delivery in CMT patients compared to the reference population. Further prospective studies on the anaesthetic management in CMT are required.

PPH rates are much lower than the general population and demonstrate the difficulties in interpreting data, based on patient recollection on a background of increasing rates of PPH and changes in estimating blood loss.^20^ An increase in PPH reported previously^9^ has not been confirmed in recent studies.^7,8^

Strengths of our study include the study being carried out in a single centre by neurologists specialised in CMT and the involvement of an obstetrician with expertise in pregnancy in patients with underlying medical conditions throughout the study from questionnaire design to result analysis. The major limitation of our study and recent similar studies is that they are all retrospective studies. The data collected in all these studies including ours is based on patients’ recollected reported outcomes as the obstetric records of these women were not available to be reviewed. Moreover, we need to be cognisant of the fact that obstetric practice has changed over time so in our study, it is difficult to have a good control population for our cohort of women who have had pregnancies over a 40-year period. Likewise, there should be caution in comparison of pregnancy outcomes in different countries where obstetric intervention rates can differ.^15^

## Conclusions

Worsening of symptoms is reported in one-third of CMT patients during pregnancy and half of the patients return to their baseline after delivery. We did not observe any significant increase in pregnancy or delivery complications and the type of delivery did not significantly differ from the normal population. From our study anaesthetic procedures were safe in comparison to the normal population and no clear evidence of complications was observed. These findings are largely reassuring for women with CMT planning pregnancy. As CMT ranges in severity, care needs to be individualised in complex cases with multidisciplinary input. Furthermore, the need for postnatal support for these mothers needs to be kept in mind.

This study and similar recent retrospective studies highlight the need for prospective studies to provide information to develop practice guidelines on the management of pregnancy and delivery in CMT. We are currently planning an international prospective study as this is the optimal way to achieve the numbers needed in a timely manner to perform such as study.

## Supplemental Material

sj-doc-1-obm-10.1177_1753495X221107328 - Supplemental material for Pregnancy and delivery in patients with Charcot–Marie–Tooth disease and related disorders Click here for additional data file. Supplemental material, sj-doc-1-obm-10.1177_1753495X221107328 for Pregnancy and delivery in patients with Charcot–Marie–Tooth disease and related disorders by Mariola Skorupinska, Gita Ramdharry, Bridgette Byrne, Matilde Laurá and Mary M Reilly in Obstetric Medicine
