# Supplementary material for: Pregnancy and delivery in patients with Charcot-Marie-Tooth disease and related disorders
Source: Obstet Med. 2022 Jun 16;16(2):83–7. doi: 10.1177/1753495X221107328 (PMC10334032; doi:10.1177/1753495X221107328)
Supplement: sj-doc-1-obm-10.1177_1753495X221107328 - Supplemental material for Pregnancy and delivery in patients with Charcot–Marie–Tooth disease and related disorders [file sj-doc-1-obm-10.1177_1753495X221107328.doc]

**Supplement 1**

**Pregnancy Questionnaire**

**Diagnosis**

Charcot Marie-Tooth Disease (CMT) ❏

Hereditary Motor Neuropathy (HMN) ❏

Hereditary Neuropathy with Liability to Pressure Palsies (HNPP) ❏

Hereditary Sensory Neuropathy (HSN) ❏

**Type of CMT**

| …………………  **CMT scoring system:**  CMT Neuropathy Score – (CMTNS) ………………….  CMT Exam Score – (CMTES) ………………………...  CMT Sensory Score – (CMTSS) ………………………   1. **Previous pregnancy questions** |
| --- |

1. Have you had any previous pregnancies?

❏ Yes ❏ No

If yes: How many pregnancy have you had? ………….

Age at first pregnancy …………………

1. How many children have you had?

………………………

1. Have you ever had a termination of pregnancy?

❏ Yes ❏ No

If yes: How many terminations have you had? ……………………

1. Have you ever had a miscarriage?

❏ Yes ❏ No

If yes: How many miscarriages have you had? …………….

At what stage of the pregnancy was your miscarriage/s? ……………..

What was the cause of your miscarriage/s?

…………………………………………

**For each pregnancy**

**Please could we ask you to answer the following questions (a separate questionnaire will be provided for each pregnancy)**

**Please state your age at each pregnancy**

**…………………………………….**

1. Did you have any genetic counselling in advance?

❏ Yes ❏ No

1. Did you consider a prenatal test?

❏ Yes ❏ No ❏ N/A

1. Were you offered **Pre-implantation Genetic Diagnosis (PGD)**

❏ Yes ❏ No ❏ N/A

1. Prior to pregnancy did you have any assistance to walk?

❏ Yes ❏ No

Type of assistance:

Orthoses:

- Shoe insert / insoles ❏
- Ankle-foot orthoses/ankle braces ❏
- Other …………………

Walking aid

- Unilateral (stick, crutch) ❏
- Bilateral (sticks/crutches, rolloter/walking frame) ❏

Wheelchair

- Intermittent use ❏
- Regular use ❏

1. Prior to pregnancy did you ever fall?

❏ Yes ❏ No

If yes: How often did you fall?

At least once a day ❏

At least once a week ❏

At least once a month ❏

At least once in 6 months ❏

At least once a year ❏

1. Prior to pregnancy did you ever experience pain?

❏ Yes ❏ No

If yes: where was the pain localised?

- Feet ❏
- Ankles ❏
- Hips ❏
- Knees ❏
- Back ❏
- Other ……………………………..

1. Prior to pregnancy did you ever feel fatigued or excessively tired?

❏ Yes ❏ No

**12.** Prior your pregnancy did you have any respiratory complications?

❏ Yes ❏ No

If yes

Were you on:

CPAP

❏ Yes ❏ No

NIPPY

❏ Yes ❏ No

Other ……….

**13.** During your pregnancy did you experience any deterioration of your symptoms related to CMT?

❏ Yes ❏ No

If yes: was this within the:

First trimester ❏

Second trimester ❏

Third trimester ❏

Within the 3 months following delivery ❏

Please indicate from the list below what symptoms you experienced:

Deterioration in walking ❏ Yes ❏ No

Falls ❏ Yes ❏ No

Pain ❏ Yes ❏ No

Worsening of balance ❏ Yes ❏ No

Worsening of hand function ❏ Yes ❏ No

**14.** During the pregnancy did you have any assistance to walk?

❏ Yes ❏ No

Type of assistance:

Orthoses:

- Shoe insert / insoles ❏
- Ankle-foot orthoses/ankle braces ❏
- Other …………………

Walking aid

- Unilateral (stick, crutch) ❏
- Bilateral (sticks/crutches, rolloter/walking frame) ❏

Wheelchair

- Intermittent use ❏
- Regular use ❏

1. During the pregnancy did you ever fall?

❏ Yes ❏ No

If yes: How often did you fall?

At least once a day ❏

At least once a week ❏

At least once a month ❏

At least once in 6 months ❏

At least once a year ❏

1. During the pregnancy did you ever experience pain?

❏ Yes ❏ No

If yes: where was the pain localised?

- Feet ❏
- Ankles ❏
- Hips ❏
- Knees ❏
- Back ❏
- Other ……………………………..

1. During the pregnancy did you ever feel fatigued or excessively tired?

❏ Yes ❏ No

**18.** During the pregnancy did you have any respiratory complications?

❏ Yes ❏ No

If yes

Were you on:

CPAP

❏ Yes ❏ No

NIPPY

❏ Yes ❏ No

Other ……….

**19.** Did you have any complications related to the pregnancy?

❏ Yes ❏ No

If yes: What problems did you have:

High Blood Pressure or pre-eclampsia ❏

Vaginal bleeding before 20 weeks ❏

Vaginal bleeding after 20 weeks ❏

Diabetes of pregnancy ❏

Urinary tract infection ❏

Preterm delivery i.e. delivery before 37 weeks ❏

Other ……………………………..

**II. Delivery**

1. What type of delivery did you have?

Natural birth ❏

Induced ❏

Assisted delivery:

Forceps ❏

Ventouse ❏

Caesarean section in labour

Emergency ❏

Elective ❏

Caesarean section before labour

Emergency ❏

Elective ❏

If you had a caesarean section, what was the reason for it?

………...…………………………………………………..

**21.** Did you see an anaesthetist to discuss pain relief during your pregnancy?

❏ Yes ❏ No

1. If you had Caesarean section what type of anaesthetic did you have?

- Epidural ❏
- Spinal ❏
- General ❏

Did you have any problems with it?

❏ Yes ❏ No

If yes: What type of problems did you have?

…………………………………………..

If you had a general anaesthetic, can you give a reason why?

……………………………………………

1. Where was your baby delivered?

- At home ❏
- Birthing centre / midwife lead ward ❏
- Labour unit ❏
- Theatre ❏
- Other …………………………..

1. Did you have any complications during delivery?

❏ Yes ❏ No

If yes: What problems did you have?

Fetal distress ❏

Slow progress ❏

Difficulty pushing the baby out ❏

Post-partum haemorrhage ❏

Blood transfusion ❏

Other …………………………..

1. Did you have pain relief during the delivery?

❏ Yes ❏ No

If yes: What type of pain relief did you have?

- Gas and air ❏
- Injections ❏
- Epidural ❏
- Tens ❏
- Other ………………………..

1. At how many weeks was your baby born …………..

Did your baby have any complications at birth?

❏ Yes ❏ No

If yes: What complications did the baby have?

…………………………………………………

**III. Post pregnancy**

1. If your symptoms related to CMT changed during the pregnancy, did they settle after delivery?

❏ Yes ❏ No

If yes how long did it take for the symptoms to settle?

………………………………………………………………………

1. Did you have any difficulties looking after the baby in the first couple of months?

❏ Yes ❏ No

If yes please detail below:

- Hand difficulties (e.g. bathing; changing and dressing) ❏
- Walking difficulties ❏
- Carrying the baby ❏
- Feeding the baby ❏
- Other…………………………..

1. If you had difficulties looking after the baby did you receive any extra help?

❏ Yes ❏ No

If yes: Who did help you?

- Partner ❏
- Relative ❏
- Live in carer (au pair) ❏
- Daily carer ❏
- Other …………………………
